# Oxymatrine Synergistically Enhances Doxorubicin Anticancer Effects in Colorectal Cancer

**DOI:** 10.3389/fphar.2021.673432

**Published:** 2021-06-30

**Authors:** Di Pan, Wen Zhang, Nenling Zhang, Yini Xu, Yi Chen, Jianqing Peng, Yan Chen, Yanyan Zhang, Xiangchun Shen

**Affiliations:** ^1^ The State Key Laboratory of Functions and Applications of Medicinal Plants (The High Efficacy Application of Natural Medicinal Resources Engineering Center of Guizhou Province), Guizhou Medical University, Guiyang, China; ^2^ The Key Laboratory of Optimal Utilization of Natural Medicine Resources, School of Pharmaceutical Sciences, Guizhou Medical University, Guiyang, China

**Keywords:** oxymatrine, doxorubicin, synergistically effect, colorectal cancer, RNA-seq

## Abstract

The combination of chemotherapy with natural products is a common strategy to enhance anticancer effects while alleviating the dose-dependent adverse effects of cancer treatment. Oxymatrine (OMT) has been extensively reported as having anticancer activity. Doxorubicin (DOX) is a chemotherapeutic DNA-damaging agent used for the treatment of carcinoma. In this study, we investigated whether synergistic effects exist with the combination treatment with OMT and DOX using human colorectal cancer cell (CRC) lines and the potential mechanisms involved in *in vitro* and *in vivo* activities. The MTT and colony formation assay results showed that compared to either OMT or DOX monotherapy, the combination of OMT + DOX markedly inhibited the growth of HT-29 and SW620 cells. Wound healing assays showed significant inhibition of cell migration with co-treatment, supported by the change in E-cadherin and N-cadherin expressions in Western blotting. Furthermore, flow cytometry analysis revealed that OMT + DOX co-treatment enhanced cell apoptosis as a result of ROS generation, whereas NAC attenuated OMT + DOX–induced apoptosis. Similarly, the apoptosis-related proteins (cleaved caspase-3, cleaved caspase-9, and the ratio of Bax/Bcl-2) were determined by Western blotting, which showed that the expressions of these markers were notably increased in the co-treatment group. Furthermore, co-administration of a low dose of DOX and OMT inhibited xenograft tumor growth in a dose-dependent manner. TUNEL assay and Ki67 staining images indicated more apoptosis and less proliferation occurred in OMT plus DOX-treated xenograft tumors. Meanwhile, the combination strategy decreased cardiotoxicity, which is the most serious side effect of DOX. RNA sequencing was performed to explore the precise molecular alterations involved in the combination group. Among the numerous differentially expressed genes, downregulated FHL-2 and upregulated cleaved SPTAN1 were validated in both mRNA and protein levels of HT-29 and SW620 cells. These two proteins might play a pivotal role involving in OMT + DOX synergistic activity. Overall, OMT in combination with DOX presented an outstanding synergistic antitumor effect, indicating that this beneficial combination may offer a potential therapy for CRC patients.

## Introduction

Colorectal cancer (CRC) is the third most common malignancy worldwide (Burnett-Hartman et al., 2021). Development of CRC is a multifactorial process with known risk factors including older age, male sex, family history, and unhealthy lifestyle including a dysregulated diet with low intake of fresh fruits and vegetables, obesity, smoking, and lack of exercise (Frank et al., 2021; Sehgal et al., 2021). As clinical therapy, the first-line chemotherapy includes fluoropyrimidines (5-fluorouracil or capecitabine) alone or combined with leucovorin (LV) as well as other cytotoxic agents, such as oxaliplatin (5-Fu/LV/oxaliplatin FOLFOX) and capecitabine/LV/oxaliplatin [CAPOX]) or irinotecan (5-Fu/LV/irinotecan [FOLFIRI]). Hence, a combination strategy is commonly used in CRC drug therapy (Chapelle et al., 2020).

**FIGURE 1:**
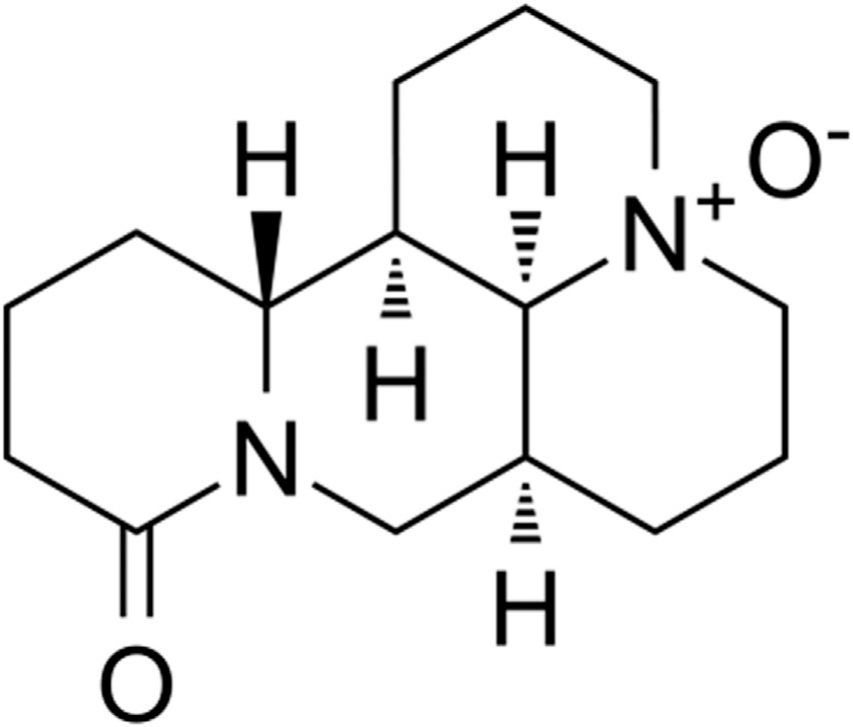
Structure of oxymatrine.

Doxorubicin (DOX), an anthracycline antibiotic, is commonly used in different types of neoplastic diseases, such as lymphomas, stomach cancer, endocrine cancer, and CRC. However, a notable incidence of cardiovascular side effects including tachycardia, hypotension, arrhythmias, and sequentially induced heart toxicity are frequently observed when used clinically (Liu et al., 2020; Galán-Arriola et al., 2021). Combination with other active drugs, especially natural products which possesses low toxicity characteristics, has become a promising strategy to alleviate DOX cardiotoxicity. Oxymatrine (OMT) (Figure 1), one of the primary alkaloids extracted from *Sophora flavescens*, has been broadly reported to be an active anticancer component in multiple types of malignancies, such as breast cancer, hepatocellular carcinoma (HCC), and non–small-cell lung cancer (NSCLC) (Halim et al., 2019; Lan et al., 2020). Furthermore, extensive studies have indicated OMT presents a synergistic effect with classic chemotherapeutic drug activity to raise drug sensitivity and reduce toxicity. For example, OMT synergistically enhances cisplatin anti-NSCLC activity by inducing antitumor immunity of CD8+ T cells (Ye et al., 2018). Further, the combination of OMT and 5-Fu improved the anticancer effect on HCC cells (Liu et al., 2016a).

In this study, we aim to investigate the anticancer effects of combination treatment with DOX and OMT in colorectal cancer *in vitro* and *in vivo*. The specific molecular regulation involving in the combination strategy was verified by RNA sequencing. Our findings provide valuable evidence of the potential therapy for OMT as a promising adjunct agent with DOX chemotherapy in colorectal cancer.

## Methods and Materials

### Cell Culture and Reagents

Human CRC cell lines HT-29 and SW620 were purchased from the Shanghai Cell Institute Country Cell Bank (Shanghai, China). HT-29 cells were cultured in McCoy’s 5A medium (Sigma, MO, United States), and SW620 cells were cultured in DMEM (Bioind, Israel). Cultures were incubated at 37°C in 5% CO_2_. OMT was purchased from Nanjing Guangrun Biological Products Co, Ltd. (Number: GR-133-151019, Jiangsu, China). DOX was purchased from Solarbio (Beijing, China).

### Cell Viability Assays

MTT assay (Solarbio Technology Co., Ltd., Beijing, China) was used to detect cell viability. Cells were trypsinized and seeded in 96-well plates (1×10^4^ cells/well) and cultured overnight and treated with various concentrations of DOX and OMT, respectively, and in combination. After treatment for 48 h, MTT (5 mg/ml) was added, and the culture was incubated for another 4 h. After removing the supernatant, 200 μl dimethyl sulfoxide (DMSO) (Solarbio Technology Co., Ltd., Beijing, China) was added. The absorbance was measured at 490 nm using multifunctional enzyme label instrument (Thermo Co., Ltd., MA, United States). The cell viability rate was calculated using GraphPad Prism v.8.0 software as follows:

[1−(control group−experimental group)/control group]×100%.

### Cell Clone Formation Assays

For colony formation analysis, HT-29 and SW620 cells (5,000 cells/well) were added to 6-well plates and treated with OMT (5 mM), DOX (0.3 μM), or OMT + DOX for 48 h. Subsequently, the medium was replaced, and the cells were cultured for an additional 14 days. The medium was changed every 2 days throughout this period. Colonies were fixed by methanol for 10 min and stained by using 0.1% crystal violet (Sigma Chemical Company, St. Louis, CA, United States) for 30 min, PBS was used for washing for three times, and then individual colonies were counted using ImageJ software.

### Synergy Determination

The data obtained from the cell viability assay were standardized to the control group. The combination index (CI) was calculated by using CalcuSyn 2.0 software program. The CI was expressed as the mean ± standard deviation (SD) of three independent experiments. The CI values represent the modes of interaction between the two drugs. CI = 1 means additive effect, CI < 1 means synergistic effect, and CI > 1 means antagonistic effect.

### DAPI Staining

HT-29 and SW620 cells were fixed using 4% paraformaldehyde for 15 min after exposure to OMT (5 mM), DOX (0.3 μM), or OMT + DOX for 48 h. Then cells were stained with DAPI (10 µg/ml) in the dark for 10 min, and then washed with phosphate-buffered saline (PBS). Fluorescence images were captured using a Leica DMi8 microscope and Leica X software (Leica, Germany) at ×400 magnification.

### Cell Apoptosis Detected by Flow Cytometry

HT-29 and SW620 cells were, respectively, treated with OMT (5 mM), DOX (0.3 μM), or OMT plus DOX for 48 h. Then the cells were washed once with PBS and trypsinized. The cells were collected by centrifugation and washed once with precooled PBS. Annexin V-FITC and/or PI (Vazyme Biotech, A211-02) was used to stain and was shielded from light for 10 min. Apoptotic cells were detected by an ACEA flow cytometer (ACEA Biosciences, Inc.) and quantified by GraphPad Prism 8.0 software.

### Reactive Oxygen Species Assay

Reactive oxygen species (ROS) levels were assayed using DCFH-DA (Beyotime Biotechnology, Shanghai, China). HT-29 and SW620 cells were exposed to OMT (5 mM), DOX (0.3 μM), or OMT plus DOX for 24 h and then washed with PBS three times. Next, cells were treated with DCFH-DA for 45 min, washed three times with PBS, and viewed under a Leica DMi8 microscope (Leica, Germany) at ×200 magnification.

### Quantitative Real-Time PCR

HT-29 and SW620 cells were, respectively, treated with OMT (5 mM), DOX (0.3 μM), or OMT + DOX for 48 h. RNA was extracted by using RNA Isolation reagent (Vazyme Biotech, R701) *via* the standard procedure. Retrotranscription was performed according to the instructions of the EasyScript^®^ One-Step gDNA Removal and cDNA Synthesis Supermix kit at 42°C for 15 min and then 85°C for 5 s. The quantitative real-time PCR (qPCR) reaction was carried out as follows: pre-denaturation at 95°C for 5 min, denaturation at 95°C for 10 s, and annealing at 60°C for 30 s, for a total of 40 cycles. The primers used for qPCR for *SPTAN1* were 5′ GCC​AAC​TCA​GGA​GCC​ATT​GTT-3′ (forward) and 5′- CGG​GTC​CGT​ATG​GTT​TCA​GAT-3′ (reverse); for *FHL-2* were 5′ TAC​AGA​CTG​CTA​TTC​CAA​CGA​G-3′ (forward) and 5′- GCA​CTG​CAT​GGC​ATG​TTG​TT-3′ (reverse); for *PPA1* were 5′ CAT​ACT​GGC​TGT​TGT​GGT​GAC-3′ (forward) and 5′- GCC​TAG​AAC​TTT​CAC​GCC​AAT-3′ (reverse); for *COX 15* were 5′ TCA​CAC​CGA​ATG​TGG​GGT​C-3′ (forward) and 5′- AGA​ACA​CGT​CCT​TTC​ATG​CCA-3′ (reverse); for *ACTB* were 5′ CAT​GTA​CGT​TGC​TAT​CCA​GGC-3′ (forward) and 5′- CTC​CTT​AAT​GTC​ACG​CAC​GAT-3′ (reverse); and for *GAPDH* were 5′ CTG​GGC​TAC​ACT​GAG​CAC​C-3′ (forward) and 5′- AAG​TGG​TCG​TTG​AGG​GCA​ATG -3′ (reverse).

### Western Blotting Analysis

In brief, HT-29 and SW620 cells were treated with OMT (5 mM), DOX (0.3 μM), or OMT plus DOX for 48 h and then lysed with lysis buffer (Solarbio Technology Co., Ltd., Beijing, China) at 4°C for 30 min. The protein concentration of each sample was quantified using a BCA Protein Assay Kit (Beyotime) according to the manufacturer’s instructions. Equal amounts of protein were separated using 6–15% SDS-PAGE gels and then transferred to polyvinyl difluoride membranes (Millipore, MA, United States). The membranes were blocked with 5% BSA in TBST at room temperature for 1 h, washed in 1×TBST for three times (10 min each), and then membranes were probed with the primary antibodies overnight at 4°C. The next day, the membrane was washed for three times with 1×TBST (10 min each), and the membranes were incubated for 1 h with secondary antibodies at room temperature. The membrane was washed for three times with 1×TBST (10 min each) following the 1-h incubation. The blots were visualized by using highly sensitive ECL Western blotting substrate (Tanon, 180-501) using a bioimaging system (Bio-Rad, ChemiDoc XRS+). The bands were quantified by using Image Lab software. Bax and Bcl-2 antibodies were purchased from ABclonal Technology (Wuhan, China); cleaved caspase-3 and cleaved caspase-9 antibodies were from Affinity Biosciences LTD. (United States); SPTAN1 antibody was obtained from Cell Signaling Technology (Danvers, MA, United States), E-cadherin, N-cadherin, and FHL-2 antibodies were purchased from Proteintech Group, Inc. (Wuhan, China); and the GAPDH antibody was from Bioworld Technology, Inc (MN, United States).

### *In Vitro* Cellular Uptake of DOX

Based on DOX autofluorescence (excitation at 488 nm and emission between 565 and 630 nm), the cellular uptake of DOX in HT-29 and SW620 cells was visualized by fluorescence microscopy and quantified by flow cytometry. Cells were seeded into 12-well plates at a density of 1×10^5^ cells/well and cultured overnight. OMT (5 mM), DOX (0.3 μM), or OMT + DOX was added to the cells for 48 h. Then the cells were washed three times with PBS, and intracellular doxorubicin uptake was observed using a Leica DMi8 microscope (Leica, Germany) at ×200 magnification.

### Wound Healing Assay

HT-29 and SW620 cells were pretreated with OMT (5 mM), DOX (0.3 μM), or OMT + DOX for 48 h, and then each group of cells was trypsinized and seeded into 12-well plates. When the cells reached more than 90% confluence after 24 h, a yellow pipette tip was used to scratch a vertical line in the adherence cells. Floating cells were washed with PBS. Images were taken with an inversion microscope at 0 and 48 h after scratching. The scratch area was measured with ImageJ program which was used to calculate the wound healing rate as follows: *A* = (A_0 h_−A_48 h_)/A_0 h_. Unit: pixel^2^.

### Tumor Xenograft Study

Male 4-to 5-week-old BALB/c nude mice (Shanghai SLAC Laboratory Animal Center, Shanghai, China) were used to establish the CRC xenograft model. HT-29 cells (1×10^6^/100 μl) were implanted by subcutaneous injected into the right axillary fossa of each mouse. After 1 week, the animals were randomly divided into seven groups (five mice per group) and given 1) control (sterile physiological saline, intraperitoneally, every other day), 2) DOX (5 mg/kg, intravenously, once every 3 days), 3) DOX (2.5 mg/kg, intravenously, once every 3 days), 4) OMT (100 mg/kg, intraperitoneally, every other day), 5) OMT (50 mg/kg, intraperitoneally, every other day), 6) OMT + DOX (100 mg/kg intraperitoneally + 2.5 mg/kg, intravenously), and 7) OMT + DOX (50 mg/kg intraperitoneally + 2.5 mg/kg, intravenously). After 24 days, the mice were sacrificed, and xenograft tumors were harvested and weighed. The tumor volume and inhibition ratio were tested. The paraffin-embedded sections (3 μm thick) were prepared and subjected to immunohistochemical analysis with the Ki-67 antibody (rabbit anti-Ki67 antibody; Proteintech Group, Inc., Wuhan, China). The apoptosis of paraffin-embedded tumor sections was detected using a TUNEL assay kit according to manufacturer’s instructions. In addition, 3-μm-thick paraffin sections of mouse hearts were prepared and stained with hematoxylin and eosin (H&E).

### RNA Sequencing Analysis

RNA-Seq by next-generation sequencing (NGS) was used to studying the transcriptome. Three independent samples from four groups of HT-29 cell cultures treated with DOX (0.3 μM), OMT (5 mM), and DOX (0.3 μM) + OMT (5 mM), and controls for 48 h were collected in TRIzol reagent (#R401-01, Vazyme, Shanghai, China). The 12 samples were transferred to a sequencing company (Sangon Biotech Co., Ltd. Shanghai) in a dry ice environment. The raw data were normalized and analyzed in software R (version: 3.6.0) along with DESeq2 packages. The selection criteria were strengthened with a threshold of false discovery rate (FDR) ≤ 0.05 and |log2FC| ≥ 1. The gene ontology (GO) analysis was performed using GOseq packages.

### Statistical Analysis

Data were expressed as the mean ± standard deviation (SD) of three independent experiments. Differences between groups were analyzed with Student’s *t* test for the *in vitro* studies and one-way ANOVA for the *in vivo* study. A *p* value < 0.05 denoted statistical significance. All statistical analyses were performed using GraphPad Prism version 8.0 software.

## Results

### OMT Synergistically Enhanced the Inhibitory Activity of DOX in CRC Cells

**FIGURE 2:**
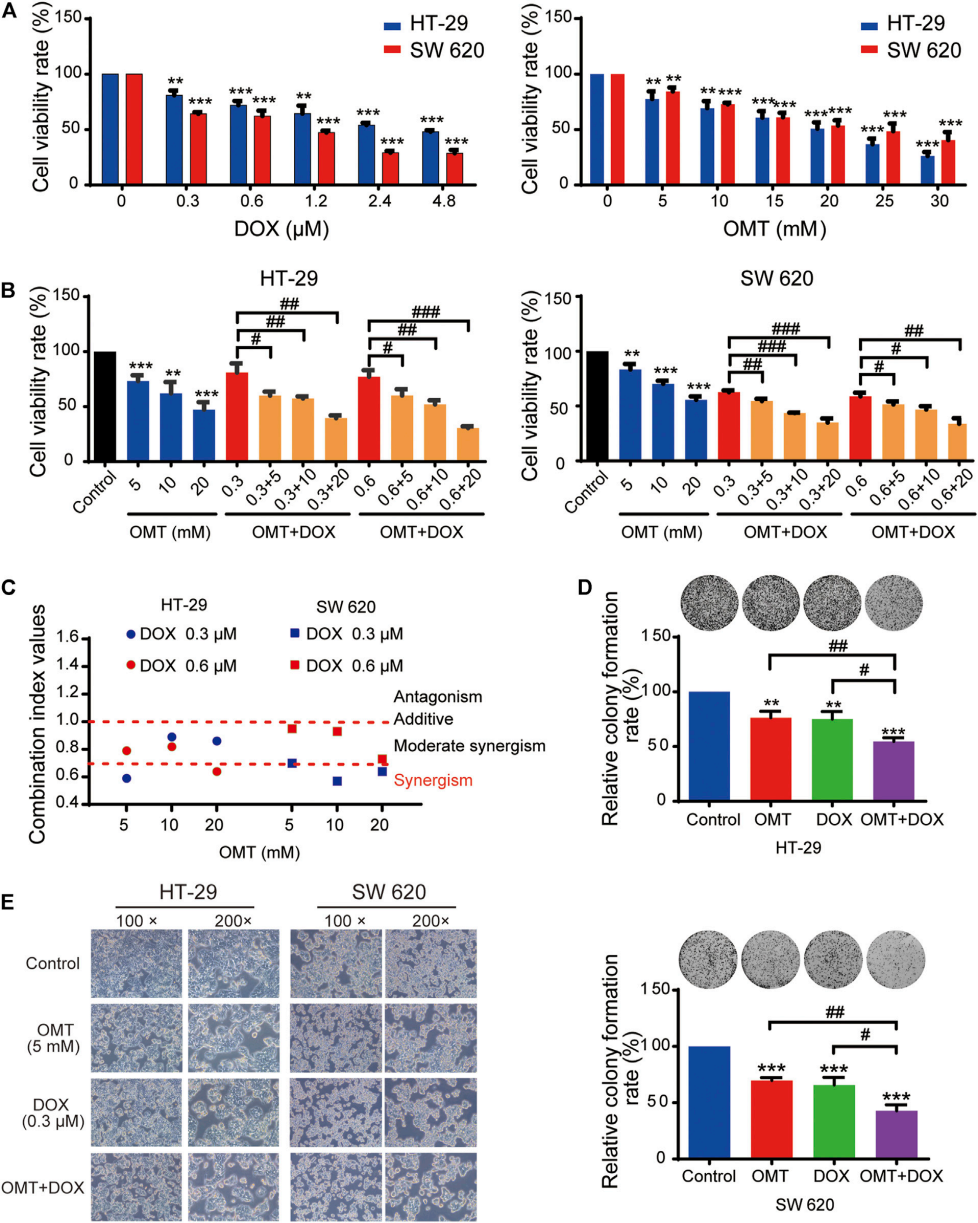
OMT synergistically enhances the antiproliferative effects of DOX in CRC cells. **(A)** The MTT assay was used to measure the cell viability of HT-29 and SW620 cells under various concentrations of OMT or DOX treatment. **(B)** HT-29 and SW620 cells viability after OMT and/or DOX treatment. **(C)** Combination index (CI) of HT-29 and SW620 cells after OMT and/or DOX treatment, CI < 1, synergistic activity; CI = 1, additive effect; CI > 1, antagonism. **(D)** Representative images of the colony formation assay in HT-29 and SW620 cells after OMT or DOX treatment **(E)** Inverted microscopy was used to observe the growth status of HT-29 and SW620 cells under OMT or DOX treatment. Data are presented as the mean ± SD of three independent experiments. **p < 0.01, ***p < 0.001 vs. the control group; ^#^ *p* < 0.05, ^##^ *p* < 0.01, ^###^ *p* < 0.001 vs. the OMT (5 mM) group, DOX (0.3 μM) group, or DOX (0.6 μM) group.

A dose–response study was performed comparing the efficacy of anticancer activity of OMT and DOX in the human CRC cell lines HT-29 and SW620. As shown in Figure 2A, in both cell lines, a dose-dependent viability inhibitory effect under OMT or DOX treatment was observed. Next, HT-29 and SW620 cells were co-treated with DOX (0.3 and 0.6 μM) and OMT (5, 10, and 20 mM), respectively. As shown in Figure 2B, each combination group presented a strikingly stronger inhibitory effect than the DOX group alone on cell activity. Next, the combination index was calculated using CompuSyn 2.0 software (Figure 2C, Table 1). The CI values of all the combination groups were less than 1, indicating the combination exerted a synergistic effect, rather than an additive effect or antagonism. The CI value of DOX (0.3 μM) combined with OMT (5 mM) was 0.59 ± 0.09 for HT-29 cells and 0.70 ± 0.08 for SW620 cells, revealing a promising synergistic inhibition capacity. The combination of DOX (0.3 μM) and OMT (5 mM) was used to subsequent experiments. HT-29 and SW620 cells were treated with DOX (0.3 μM) or/and OMT (5 mM) for 48 h, and cell density and morphology were observed. As shown in Figure 2E, after drug treatment, the cell density decreased. Furthermore, the cell colony formation assay was performed to test cell proliferation. The results showed that co-treatment with OMT and DOX notably reduced the formation of colony formation in both cell lines (Figure 2D). Collectively, these results indicated that OMT enhanced the inhibitory activity of DOX in CRC cells.

**TABLE 1:** Combination index (CI) of HT-29 and SW620 cells.

DOX (μM)	OMT (mM)	CI ± SD	
		HT-29	SW620
0.3	5	0.59 ± 0.09	0.70 ± 0.08
	10	0.89 ± 0.07	0.57 ± 0.01
	20	0.86 ± 0.08	0.64 ± 0.10
0.6	5	0.79 ± 0.25	0.95 ± 0.15
	10	0.82 ± 0.13	0.93 ± 0.13
	20	0.64 ± 0.04	0.73 ± 0.18

### Effects of OMT and DOX on Apoptosis, Metastasis, and Cellular Uptake of DOX in CRC Cells

**FIGURE 3:**
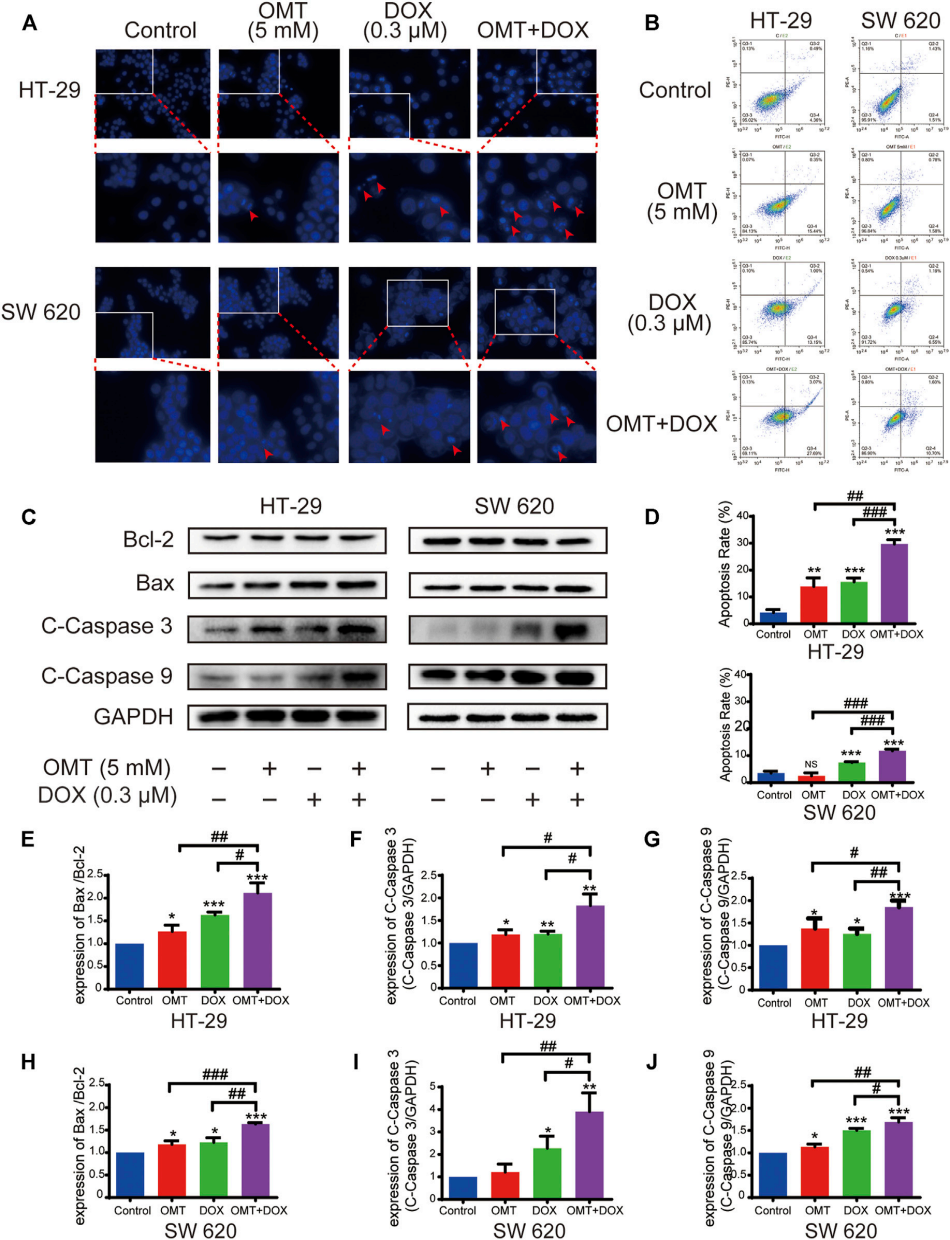
OMT combined with DOX induced apoptosis of CRC cells. **(A)** DAPI staining (blue) was used to observe the changes in the nucleus of HT-29 and SW620 cells under OMT or DOX treatment. **(B)** Cell apoptosis was detected by Annexin V-FITC/PI binding assay after OMT and/or DOX treatment in HT-29 and SW620 cells. **(C)** Western blotting was used to analyze the expression of Bcl-2, Bax, cleaved caspase-3, and cleaved caspase-9. **(D)** Statistical analysis of total apoptosis rates including early and late apoptosis (Q2 + Q4, respectively). **(E–J)** The densitometric analysis of protein bands was performed and normalized with the corresponding GAPDH content. Data are presented as the mean ± SD of three independent experiments. *p < 0.05, **p < 0.01, ***p < 0.001 vs. the control group; ^#^ *p* < 0.05, ^##^ *p* < 0.01, ^###^ *p* < 0.001 vs. the OMT (5 mM) group or DOX (0.3 μM) group.

DAPI staining demonstrated that morphological changes occurred in the cells treated with OMT or DOX, and more obvious typical apoptotic morphological changes were observed in the OMT + DOX group (Figure 3A, arrow). Next, we quantified the apoptosis rates following OMT and DOX treatment using a flow cytometry assay. The results showed that co-treatment with OMT and DOX caused greater apoptosis than exposure to either OMT or DOX alone in both HT-29 and SW620 cell lines (Figures 3B,D). To further detect the apoptosis effects induced by OMT or/and DOX, we assessed the expression of apoptosis-related proteins cleaved caspase-3, cleaved caspase-9, and the ratio of Bax/Bcl-2 by Western blotting. The results demonstrated that co-treatment with OMT and DOX significantly enhanced the expression of those proteins in HT-29 and SW620 cells (Figures 3C,E–J).

**FIGURE 4:**
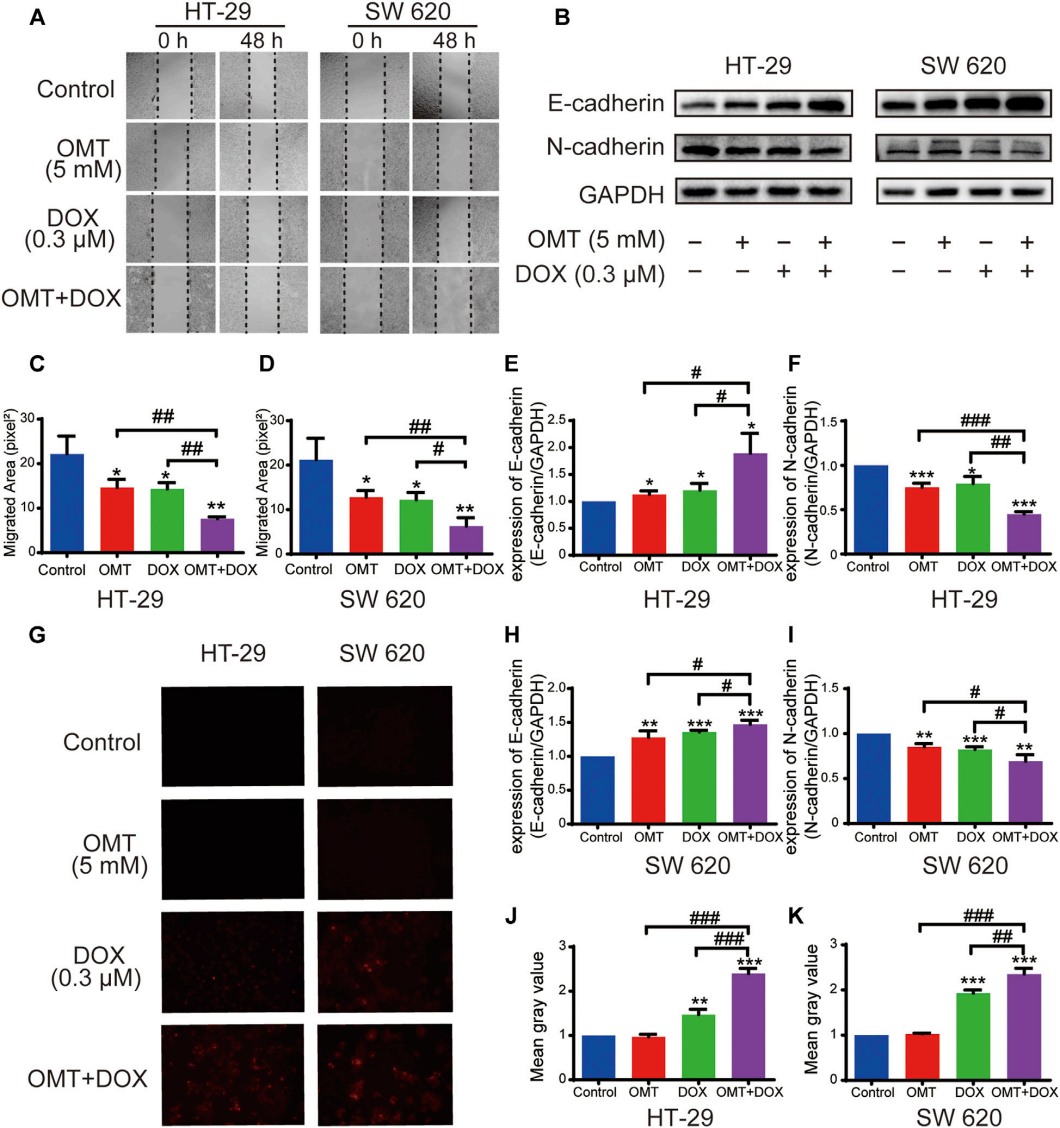
OMT combined with DOX inhibited migration of CRC cells. **(A)** Wound healing assay of HT-29 and SW620 cells following treatment with OMT or/and DOX. Images were captured at 0 and 48 h after wounding the cell layer (magnification, ×50). **(B)** Western blotting assay was used to analyze the expression of E-cadherin and N-cadherin. **(C,D)** The wound healing rate of HT-29 and SW620 cells was expressed as percentage of the scratch area. **(E,F)** The densitometry analysis of each protein was performed and normalized with the corresponding GAPDH content. **(G)** Uptake of Dox in HT-29 and SW620 cells was observed under fluorescence microscopy. The red fluorescence indicates DOX. **(H,I)** The densitometry analysis of each factor was performed and normalized with the corresponding GAPDH content. **(J,K)** ImageJ was used to calculate the average fluorescence intensity of DOX. Data are presented as the mean ± SD of three independent experiments. *p < 0.05, **p < 0.01, ***p < 0.001 vs. the control group; ^#^ *p* < 0.05, ^##^ *p* < 0.01, ^###^ *p* < 0.001 vs. the OMT (5 mM) group or DOX (0.3 μM) group.

Next, we speculated that combination treatment might weaken cell migration in HT-29 and SW620. The wound healing assay was utilized to show that the migration effect was significantly suppressed when CRC cells were exposed to both OMT and DOX (Figures 4A,C–D). In addition, we found the E-cadherin expression was upregulated, while N-cadherin expression was downregulated under OMT + DOX (Figures 4B,E–F,H–I), revealing that combination use could suppress CRC cell epithelial–mesenchymal transition (EMT) characteristics. Moreover, based on the spontaneous red fluorescence of DOX, the cellular uptake of DOX could be detected using fluorescence microscopy. As shown in Figures 4G,J–K, OMT enhanced the level of fluorescence produced by DOX, suggesting that OMT promoted CRC cells to capture more DOX. Taken together, these results further supported the combination of OMT + DOX to reduce CRC proliferation and metastasis, while increasing DOX cellular absorption.

### OMT Synergistically Enhanced DOX-Induced ROS Generation

**FIGURE 5:**
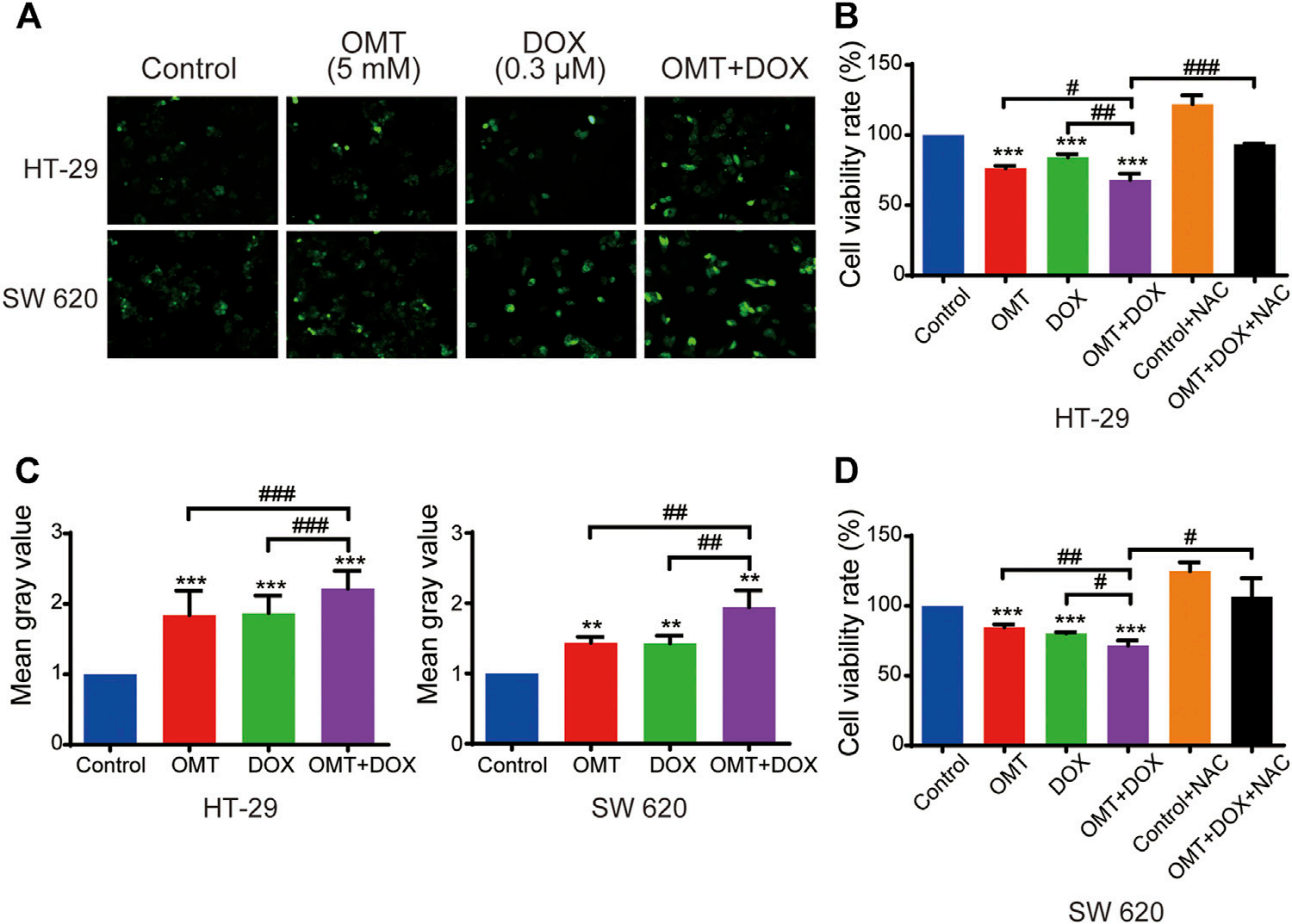
OMT and DOX act synergistically to upregulate ROS levels in CRC cells. **(A)** Intracellular ROS detection after treatment with OMT, DOX, or OMT plus DOX. **(B)** Analysis of cell viability after exposure to OMT and/or DOX in the presence or absence of NAC. **(C)** ImageJ was used to calculate green fluorescence intensity. **(D)** Analysis of cell viability after dealt with OMT and/or DOX in the presence or absence of NAC. Data are presented as the mean ± SD of three independent experiments. **p < 0.01, ***p < 0.001 vs. the control group; ^#^ *p* < 0.05, ^##^ *p* < 0.01, ^###^ *p* < 0.001 vs. the OMT (5 mM) group, DOX (0.3 μM) group, or OMT plus DOX.

We next investigated intracellular ROS levels in HT-29 and SW620 cells treated with OMT and/or DOX using the DCFH-DA probe. As shown in Figures 5A,C, OMT or DOX treatment increased ROS generation in HT-29 and SW620 cells compared to the control group, and the effect was much more pronounced in the co-treatment group (*p* < 0.0001, *p* < 0.001, respectively). To further confirm the important role of ROS in the anticancer effect induced by the combination of OMT and DOX, NAC was added to deplete intracellular ROS levels. The combined effect was reversed on diminishing ROS levels, suggesting that ROS may play a pivotal role in the synergetic mechanism induced by OMT and DOX (Figures 5B,D).

### OMT Facilitated the Anticancer Effects of DOX *In Vivo*

**FIGURE 6:**
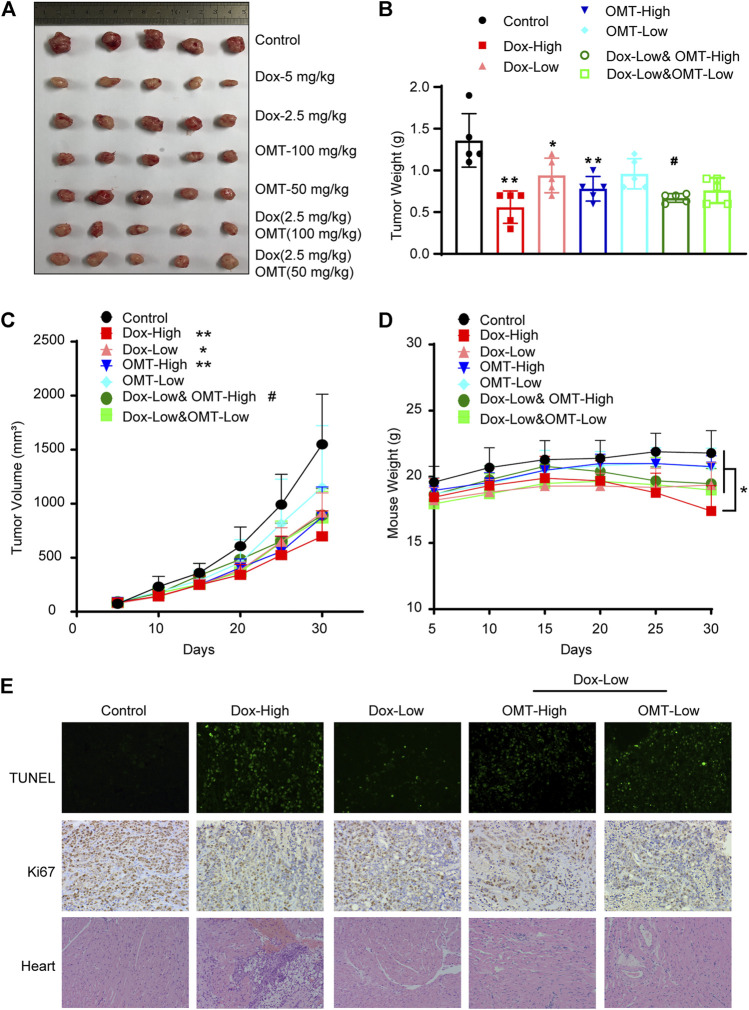
Effects of OMT and DOX on tumor growth in a CRC xenograft model. A CRC mouse model was established by introducing a xenograft of HT-29 cells. The mice were treated with saline (control), OMT, Dox, or OMT plus DOX. **(A)** Image of excised tumors at the conclusion of the experiment. **(B)** Weight of different groups of tumor tissue. **(C)** Tumor volume progression as a function of time. **(D)** Mouse weight as indicated over time. **(E)** Representative images of hematoxylin and eosin staining on cardiac tissue sections, TUNEL assay and immunohistochemical staining with antibodies to Ki67 on tumor tissue sections. *p < 0.05, **p < 0.01 vs. the control group or DOX (5 mg/kg); ^#^ *p* < 0.05, vs. the DOX (2.5 mg/kg) group.

A subcutaneously implanted tumor model was established to evaluate the combined anticancer effects observed *in vitro*. Although a high dose of DOX (5 mg/kg) presented a striking inhibitory effect on xenograft tumors, it also produced a statistical reduction of mice weight, suggesting high-dose DOX caused a serious side effect. Administration alone with a dose of OMT (100 mg/kg) also exhibited a promising anticancer effect, inducing a 43.00 ± 8.55% inhibitory rate. Furthermore, co-treatment with 100 mg/kg OMT and 2.5 mg/kg DOX enhanced the anticancer activity (52.14 ± 3.32%) and ameliorated weight loss (Figures 6A–D). Next, the xenograft tumor tissues were examined by TUNEL assay and Ki67 staining. In the OMT and DOX group, the proliferation was decreased and apoptosis was increased when compared to the DOX monotherapy group assessed using the TUNEL assay. Furthermore, an increase in inflammatory cell infiltration and myocardial fiber denaturation, and edema were observed in H&E staining heart tissues from the high-dose DOX treatment group. Instead, the OMT + DOX combination group presented a normal histomorphology, revealing that OMT could diminish the severe toxicity of DOX (Figure 6E). Taken together, these results demonstrated that OMT facilitated the anticancer effects of DOX *in vivo*, and weakened cardiac damage induced by DOX.

### Bioinformatics Analysis of the RNA-Seq Assay

**FIGURE 7:**
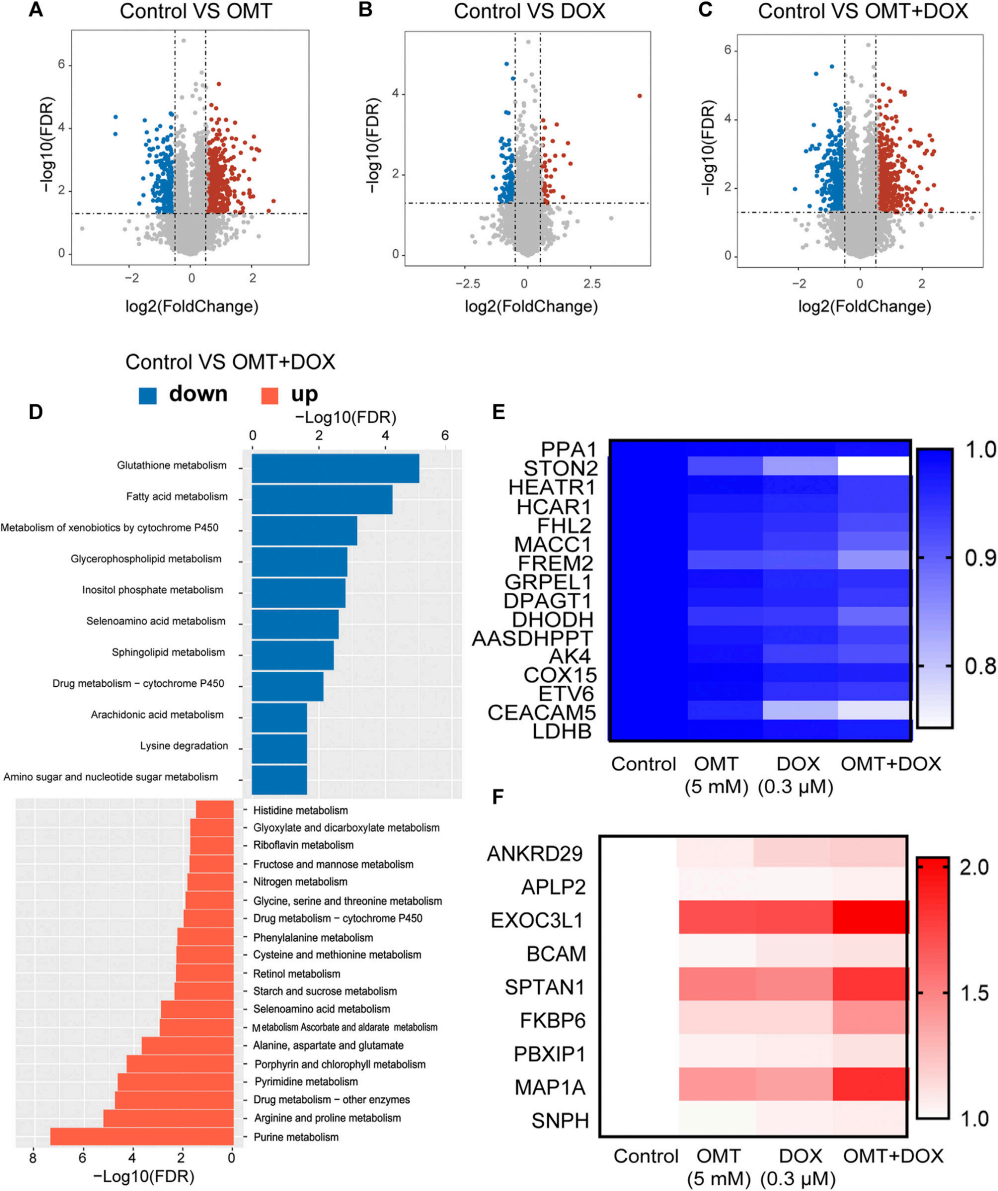
RNA-seq analysis of mRNA expression of OMT and DOX in HT-29 cells. **(A–C)** Volcano map showing changes in mRNA levels after OMT and/or DOX treatment. **(D)** Gene ontology (GO) analysis was utilized to evaluate the canonical pathways in the combination treatment group. **(E,F)** Heatmap analysis of upregulated or downregulated genes after OMT and/or DOX treatment.

To further explore the molecular mechanism involved in the combination effect of DOX and OMT in CRC cells, an RNA-Seq assay was performed. As shown in Figures 7A–C, three volcano plots were developed to illustrate the differential expression genes (DEGs) according to the FDR and Log2FC values. Compared to the control group, a single use of DOX generated 419 upregulated and 599 downregulated genes; a single use of OMT generated 2044 upregulated and 1,225 downregulated genes, while the combination group generated 1,979 upregulated and 1,503 downregulated genes. Next, gene ontology (GO) analysis was utilized to evaluate the canonical pathway activated by the combination treatment. The GO enrichment analysis revealed that among the downregulation DEGs were those involved in glutathione, fatty acid, and cytochrome P450 metabolism, while the upregulated DEGs were those related to purine, arginine, and proline metabolism (Figure 7D). Next, we analyzed the overlapping genes among those up- or downregulated, respectively, and chose several genes whose changes were much stronger in the combination group than in single use of OMT or DOX. As shown in Figure 7E,F, these genes were selected from thousands of data items and suggest they play a pivotal role in inducing the molecular mechanism of the OMT and DOX combination.

### OMT Enhanced the DOX Sensitivity by Decreasing FHL-2 and Increasing SPTAN1 Cleavage

**FIGURE 8:**
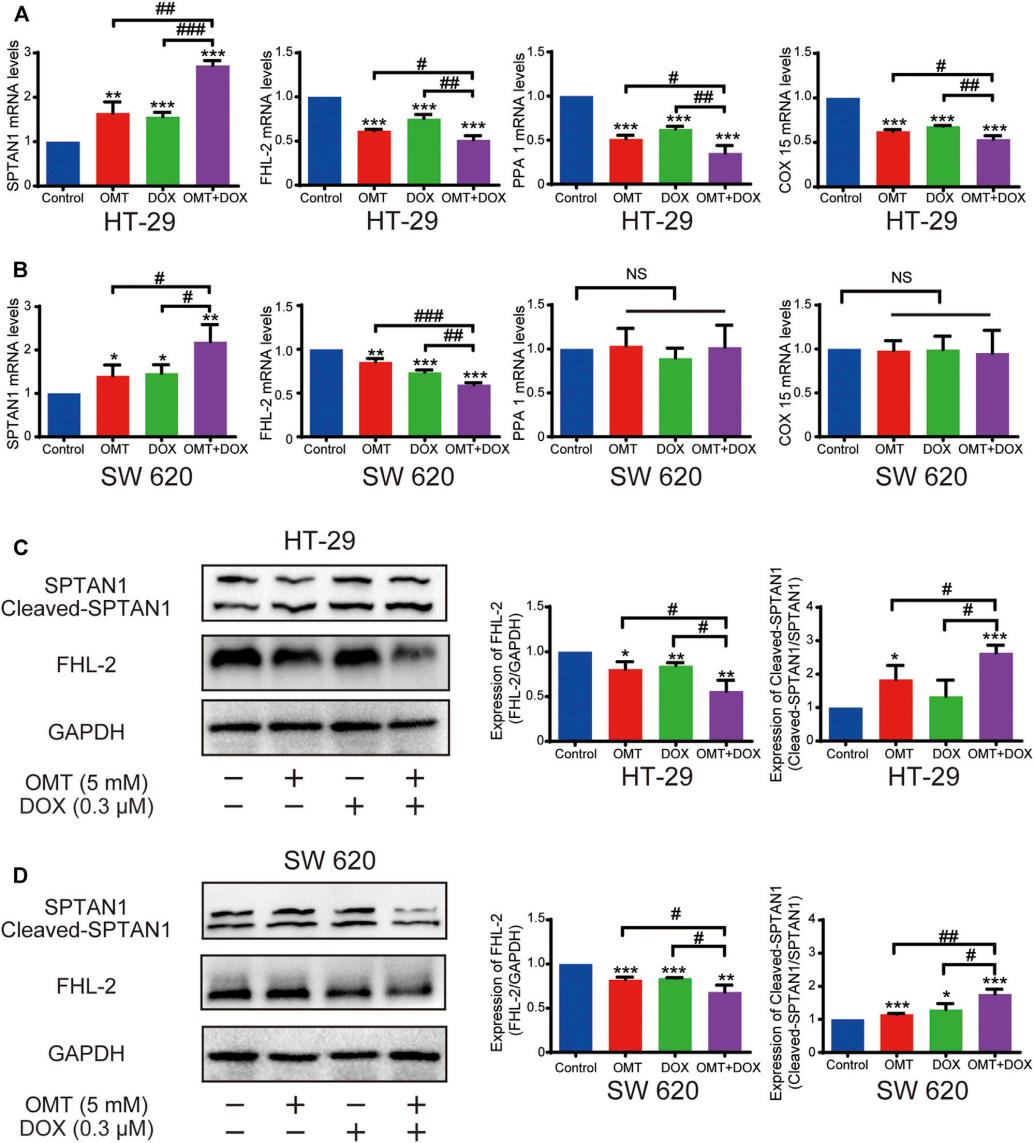
Effects of OMT and DOX on FHL-2 and SPTAN1 in CRC cells. **(A,B)** mRNA expression of SPTAN1, FHL-2, PPA1, and COX 15 on HT-29 and SW620. HT-29 **(A)** and SW620 **(B)**. **(C–D)** Western blotting assay was used to analyze the protein expression level of SPTAN1, cleaved-SPTAN1, and FHL-2 in HT-29 and SW620 cells; the densitometric analysis of every factor was performed and was normalized with the corresponding GAPDH content. Data are presented as the mean ± SD of three independent experiments. *p < 0.05, **p < 0.01, ***p < 0.001 vs. the control group; ^#^ *p* < 0.05, ^##^ *p* < 0.01, ^###^ *p* < 0.001 vs. the OMT (5 mM) group or DOX (0.3 μM) group.

To identify changes in expression of genes associated with the apoptosis pathway, we selected four genes (*SPTAN1, PPA1, COX15,* and *FHL-2*) and validated their expression in HT-29 and SW620 cells. The mRNA expression of these four genes was confirmed in HT-29; however, only *SPTAN1* and *FHL-2* were validated in SW620 cells (Figures 8A,B). Further, in line with qPCR results, FHL-2 protein expression was decreased after a single use of DOX or OMT and was much lower in the combination group. Similarly, the cleaved SPTAN1, the activated form for apoptosis, was significantly increased in the combination group, indicating that co-treatment of DOX and OMT enhanced apoptosis by inducing SPTAN1 cleavage (Figures 8C,D). Nonetheless, the protein levels of PPA1 and COX 15 were validated only in HT-29. Taken together, these data revealed that FHL-2 and SPTAN1 might be the key roles in the combination effect of DOX and OMT on CRC cells.

## Discussion

CRC is a major gastrointestinal carcinoma that affected approximately 1.8 million people worldwide in 2018 (Liu et al., 2019). Traditional treatments such as surgery, radiation therapy, and chemotherapy are associated with multiple side effects, and the outcomes of CRC therapies are far from satisfactory (Liu et al., 2019). Currently, many studies have revealed that combination therapy with natural plants and chemotherapeutic drugs could enhance anticancer effects, reduce therapy resistance, and decrease toxicity (Liu et al., 2018). In the present study, we determined that OMT and DOX combination significantly suppress CRC cell growth, induce cell apoptosis, and display a magnificent chemo-sensitization effect *in vitro* and *in vivo*. Therefore, it represents a promising approach to application of the combination of OMT and DOX for clinical treatment for CRC.

DOX is the most effective anticancer agent, and it has been used mainly for the treatment of solid tumors in children, soft tissue sarcomas, and aggressive lymphomas. However, the incidence of DOX-induced cardiotoxicity (DIC) has greatly limited its clinical application (Tian et al., 2021). There are two main strategies under study to alleviate DOX cardiotoxicity: 1) structural modifications by pharmaceutical or chemical methods of the parent DOX, such as the synthesis of DOX analogues (idamycin and epirubicin) or development of new DOX formulations (liposome DOX); and 2) drug combinations being evaluated in pharmacological research. At present, dexrazoxane (ADR-529, ICRF-187), a cyclic derivative of ethylenediaminetetraacetic acid, is the only agent approved by the U.S. Food and Drug Administration (FDA) to reduce DOX-induced DIC (Yarmohammadi et al., 2021). However, its clinical use has been restricted due to potential carcinogenic risks. Accordingly, seeking novel combination drugs has become an effective strategy to alleviate DIC, especially natural compounds due to their activity and reduced toxicity, such as myricitrin (Sun et al., 2016), resveratrol (Tian et al., 2020), *β*-caryophyllene (Meeran et al., 2019), and punicalagin (Ye et al., 2019). OMT is a quinolizidine alkaloid compound extracted from the root of *Sophora flavescens*. Current studies have shown that OMT has significant antitumor effects, among which it can inhibit the proliferation and EMT, and it can induce apoptosis of CRC cells. Previously, OMT was been reported to ameliorate cardiovascular diseases, such as heart failure, hypertrophy of heart ventricles, myocardial injury induced by ischemia, and ventricular arrhythmia (Zhang et al., 2017; Jia et al., 2019; Wu et al., 2019). In this study, we revealed that OMT not only induced CRC cell inhibition but also presented a synergetic effect of DOX activity against CRC. In our mice model, although a 5 mg/kg dose of DOX treatment induced notable tumor inhibition, weight loss and myocardial damage were observed in treated mice, indicating such dosage was unsuitable. However, a 2.5 mg/kg DOX dose is not sufficient against CRC. Hence, we proposed the strategy of 2.5 mg/kg DOX combined with OMT (100 mg/kg and 50 mg/kg) administration to increase the inhibitory effects and decrease side effects. Intriguingly, our results presented the combination strategy is a promising approach to CRC treatment. However, based on the body surface area, the human equivalent dose should be 0.081-fold that of mice (Nair and Jacob, 2016). For an adult of 70 kg weight, to achieve 100 mg/kg OMT, the dose might be 0.567 g, which is very high for clinical use. Owing to its promising activity, we considered that further studies should concern about the pharmaceutical modification of OMT. A limitation of our study is that we were unable to define the specific role for OMT in alleviating cardiac injury as we did not provide any results to demonstrate the beneficial effect and mechanisms involving OMT. Further investigation into the potential myocardial protective role of OMT is warranted.

We performed RNA-seq using HT-29-treated cells and analyzed transcriptome variations under exposure to OMT and DOX treatment. Strikingly SPTAN1 and FHL-2 were identified as potential targets for combination treatment. FHL-2, or the four and a half LIM domains 2, is a protein containing only four and a half LIM domains in its molecular structure, and plays an important role in transcriptional regulation, apoptosis, and the occurrence and development of tumors (Cao et al., 2015). The role of FHL-2 in tumors is bidirectional, whereby overexpression of FHL-2 has been observed in colorectal, gastric, and pancreatic cancer, while it has been reported to be downregulated in HCC (Verset et al., 2016). FHL-2 is highly expressed in primary CRC and promotes the proliferation, invasion, and metastasis of CRC cells (Zhang et al., 2010; Hua et al., 2016). Our results showed that both OMT and DOX could decrease the protein levels of FHL-2 in HT-29 and SW620 cells, and the decrease was more significant after treatment with the combination of OMT and DOX. Hence, we speculated that FHL-2 might be a promising target for combination treatment.

In addition, we find that OMT and DOX increased cleaved SPTAN1 (non-erythroid spectrin II) protein levels, and the combination was further enhanced, revealing that SPTAN1 might be the critical important target. SPTAN1 is an important cytoskeletal protein that is also involved in cell adhesion, cell-to-cell contact, and apoptosis. Overexpression of SPTAN1 in cancer was first described in sporadic CRC (Ackermann et al., 2019). Intriguingly, increased cytoplasmic SPTAN1 levels were detectable not only in colon carcinoma but also in Crohn’s disease and in other epithelial neoplasms, including adenocarcinomas of the stomach and small intestine, suggesting enhanced SPTAN1 levels as a nonspecific marker for neoplasia of both benign and malignant origins (Ackermann and Brieger, 2019). A recent study also described a positive correlation with metastasis in colorectal cancer (Ackermann et al., 2019). The cleavage of SPTAN1 occurs during apoptosis and can be used as a marker of cancer therapeutic efficacy (Ackermann and Brieger, 2019). These data suggest that FHL-2 and SPTAN1 play a key role in the molecular mechanism of OMT and DOX combination anticancer activity. However, in this study, we did not study how FHL-2 and SPTAN1 act as the specific targets under OMT and DOX treatment. A gain or loss of functions for the two targets should be performed to illustrate their combined effects in future studies.

In conclusion, our study shows that the combination of DOX and OMT exerted superior synergistic effects on CRC cells *in vitro* and *in vivo* than either DOX or OMT alone, and the synergistic mechanism may be achieved by FHL-2 and SPTAN1. Our study provided preclinical evidence that the combination treatment with OMT and DOX could be a novel and promising therapeutic approach to the treatment of colon cancer, which warrants further investigation in a clinical setting.

## Data Availability

The original contributions presented in the study are included in the article/supplementary material, further inquiries can be directed to the corresponding author.
